# Serum Biomarkers in Restless Legs Syndrome: Beyond the Classical Iron Paradigm—A Scoping Review

**DOI:** 10.3390/ijms27083385

**Published:** 2026-04-09

**Authors:** Krasimir Avramov, Todor Georgiev, Aneliya Draganova, Kiril Terziyski

**Affiliations:** Department of Pathophysiology, Medical University–Plovdiv, 4002 Plovdiv, Bulgaria; tgeorgiev@pathophysiology.info (T.G.); adraganova@pathophysiology.info (A.D.); kterziyski@pathophysiology.info (K.T.)

**Keywords:** restless legs syndrome, biomarker, hepcidin, inflammation, high-throughput omics, cytokines, C-reactive protein, oxidative stress

## Abstract

Restless legs syndrome (RLS) is one of the most prevalent sleep disorders, yet its diagnosis continues to rely almost entirely on subjective symptom descriptions. This persistent dependence on phenomenology reflects the absence of reliable biological markers to aid in the process of diagnosis or monitoring. However, there is accumulating molecular evidence that suggests that RLS is associated with systemic biological alterations. These extend beyond the traditional paradigm of iron deficiency. The present scoping review synthesizes the current research on circulating serum biomarkers investigated in RLS outside classical iron indices. A comprehensive search of PubMed, Scopus, and Web of Science databases identified 1050 records, of which 50 studies met eligibility criteria and were included. In the processing of data, clusters emerged into several recurring biological domains, including dysregulated iron regulatory signaling (hepcidin), low-grade immune activation, oxidative stress, and neuroaxonal injury markers. High-throughput omics studies reveal molecular network perturbations involving inflammatory pathways, complement activation, metabolic signaling, and cellular stress responses. Biomarker associations appear stronger when linked to objective motor burden. These findings suggest that RLS may involve multifarious molecular changes detectable in the serum. Consequently, this can support the transition from symptom-based diagnosis toward biomarker-informed stratification, which may enable more precise disease characterization and improved diagnostic accuracy.

## 1. Introduction

Restless legs syndrome (RLS) is the most prevalent neurological motor sleep disorder, and it carries a substantial burden due to its high prevalence and marked impact on the quality of life [1,2,3]. Despite decades of research, its pathophysiology remains only partially understood, with emerging evidence continuing to add layers of neurobiological complexity [4,5,6]. Nowadays, it is acknowledged that a multifaceted neurotransmitter dysfunction is present in RLS patients—involving perturbations in dopamine, glutamate and adenosine pathways [7,8,9]. Strong evidence suggests compartmentalized iron metabolism dysfunction affecting structures in the central nervous system [5,6,10]. The latter is hypothesized to be at the root of neurotransmitter abnormalities, but firm evidence is lacking to establish a sound theory [11].

**Figure 1:**
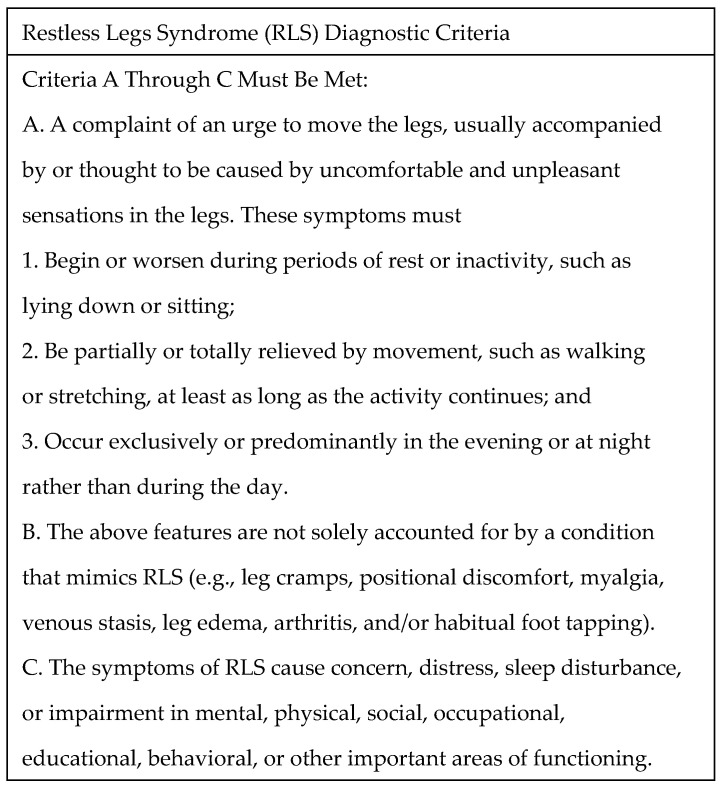
Diagnostic criteria of restless legs syndrome by *International Classification of Sleep Disorders*, Third Edition, Text Revision 2024.

However, in contrast to those advances, the diagnostic framework has changed remarkably little since the seminal clinical descriptions by Karl-Axel Ekbom [8,12,13]. This reliance primarily on phenomenology in the clinical criteria is still reflected in *The International Classification of Sleep Disorders*, Third Edition, Text Revision 2024 [14] (Figure 1).

There exists an underrecognition of this disorder, which partially stems from the heterogeneous clinical presentation of restless legs syndrome [6,15,16]. Symptoms may vary widely in intensity, frequency, anatomical distribution and descriptors the patients use [17]. Often, symptomatology overlaps with other neurological, vascular, and sleep-related conditions, making the disorder difficult to recognize and classify within traditional disease frameworks [18]. The absence of consistent objective findings on routine examination or standard laboratory testing has further contributed to the diagnostic uncertainty, clinical neglect, and, most importantly, skepticism [15,19]. This acts as a source of inconsistency in the epidemiological data, which highlights limitations in the diagnostic criteria [2,20,21]. In 2015, Carlos et al. showed in their study of newly graduated physicians that the diagnosis can be distorted by semantics alone—simply calling the condition “Restless Legs Syndrome” inflated self-diagnosis nearly seven-fold compared to “Willis–Ekbom Disease” [22]. The persistence of a symptom-based diagnostic approach represents a double-edged sword for sleep specialists. On one hand, it allows rapid, accessible diagnosis marked by technological independence. On the other hand, it creates substantial vulnerability to overdiagnosis, as the defining features of RLS describe a behavioral and sensory phenotype [17].

There is a clear movement toward biologically associated diagnostic models in various medical fields. Sleep medicine—and particularly RLS—now faces the same pressure to transition from descriptive syndromic classification toward objective, mechanism-informed stratification. Within the RLS diagnostic and therapeutic framework, the only objective variables measured are periodic leg movement index (PLMI) and ferritin concentration or total iron-binding capacity [4,23,24,25]. The former is assessed by a polysomnographic study, while the latter is assessed by clinical laboratory measurement. None of them is included in the official criteria for diagnosis [14]. Lately, ferritin levels have been incorporated into therapeutic algorithms, but the threshold used for decision-making is arbitrary rather than evidence-based [24,25]. While there is growing support for the theory of iron dysmetabolism and not simply iron deficiency being at the core of the pathogenesis of restless legs syndrome, sleep specialists traditionally continue to use only classical assessment of the iron status with the above-mentioned parameters [24]. These are incapable of a satisfactory description of the iron compartmentalization and overall metabolism [26].

It is widely anticipated that the era of high-throughput omics technologies will provide meaningful answers to the search for objective biological markers in complex neurological disorders. Restless legs syndrome, long-defined exclusively by clinical phenomenology, represents a prime candidate for such advances. Over the past two decades, a burgeoning body of research has explored circulating biomarkers that may reflect systemic processes linked to RLS pathogenesis.

Despite this expanding interest, the overall number of biomarker studies in RLS remains modest. The existing literature is also marked by substantial methodological heterogeneity. Differences in study design, patient selection, diagnostic criteria, disease severity assessment, comorbidity profiles and laboratory techniques make direct comparison across studies burdensome.

Previous reviews have predominantly examined isolated biomarker domains, particularly cytokines or banal inflammatory markers, often without integrating these findings into a unified biological framework. The present review addresses this gap by synthesizing evidence across multiple molecular domains—including iron regulation, immune activation, oxidative stress, and omics-derived signatures. By this, we attempt an identification of converging biological patterns and their relationship to clinical phenotypes.

The aim of the present scoping review is therefore to examine what advances have been achieved in the search for reliable serum biomarkers for restless legs syndrome. Our intention is to explore the research that is focused on molecules and signatures outside of the classical iron status measurements (serum iron, ferritin, and total iron-binding capacity). By synthesizing the breadth of existing evidence rather than focusing narrowly on effect size or meta-analytic pooling, this review seeks to characterize the current scope of circulating biomarker research in RLS and to identify directions for future lines of research.

To achieve the aim of the article, the researchers adopted the definition for “biomarker” given by the FDA-NIH Biomarker Working Group. In their continuously updated online document “Biomarkers, EndpointS, and other Tools” (BEST), biomarkers are described in relation to their ability to diagnose, monitor, predict, participate in prognosis, etc. Specifically, we focused on serum biomarkers with potential diagnostic or monitoring capability, while recognizing the definition by BEST for such—“a biomarker used to detect or confirm presence of a disease or condition of interest or to identify individuals with a subtype of the disease” [27].

## 2. Methods

### 2.1. Search Strategy

This scoping review was conducted in accordance with the PRISMA Extension for Scoping Reviews (PRISMA-ScR) and methodological guidance from the Joanna Briggs Institute (JBI) [28,29]. A structured, multi-step search strategy was used to identify studies investigating circulating serum or plasma biomarkers in restless legs syndrome. We formulated the following JBI-compliant review question: *What circulating serum biomarkers beyond the classical iron indices have been investigated in patients with restless legs syndrome, and what biological domains and clinical associations have been identified?*

The primary search string used across databases was: (“restless legs syndrome”) AND (“biomarker” OR “serum marker” OR “plasma marker” OR “inflammatory marker” OR “iron metabolism” OR “cytokines” OR “proteomics” OR “metabolomics”). The Boolean operator AND was used to ensure inclusion of studies specifically related to restless legs syndrome, while the operator OR was used to capture studies investigating a broad range of biomarker classes, including inflammatory, iron-related, proteomic, and metabolomic biomarkers. In the databases search engines additional rule was added when it was applicable—“restless legs syndrome” to be present in the title or abstract.

The comprehensive search was conducted across MEDLINE (PubMed), Web of Science and Scopus. In line with the exploratory nature of a scoping review and the aim to map the full development of the field, the search was not restricted by year of publication and covered all records from database inception to the final search date—30 January 2026. Only articles published in English were included.

### 2.2. Eligibility and Selection of Studies

All records identified through the search were imported into reference management software, and duplicates were removed. Titles and abstracts were screened for relevance to the review question, followed by full-text assessment of potentially eligible articles. Study selection was guided by predefined inclusion and exclusion criteria.

Studies were eligible for inclusion if they involved human participants diagnosed with RLS according to established clinical criteria by the International Classification of Sleep Disorders and reported quantitative measurements of circulating biomarkers in serum or plasma. Both primary (idiopathic) and secondary forms of RLS were considered eligible. Observational and interventional study designs were considered if they presented original data on serum or plasma biomarker levels in individuals with RLS, either in comparison with control groups or in relation to disease severity, phenotype, or treatment response.

**Figure 2:**
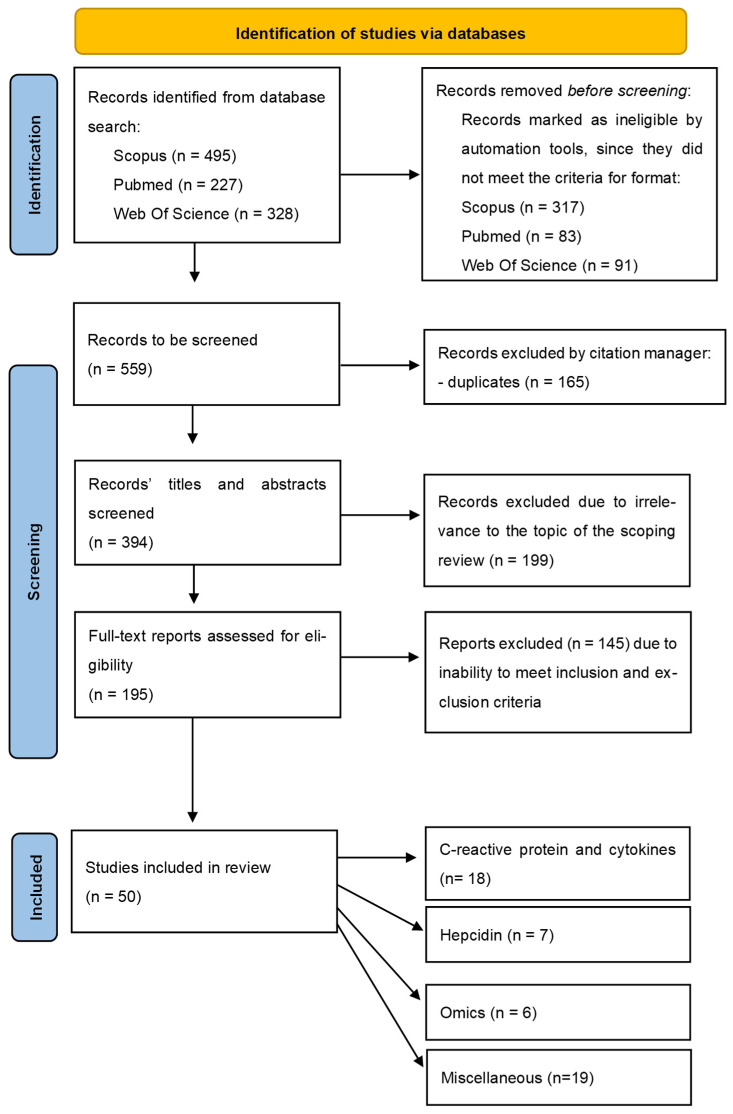
The PRISMA-ScR flow chart, displaying the multistep process of database search and selection of studies to be included in the scoping review.

Studies were excluded if they examined biomarkers exclusively in cerebrospinal fluid, tissue, or through imaging modalities without blood-based measurements. Articles focusing solely on genetic polymorphisms or gene expression without corresponding circulating protein or metabolite data were also excluded. Review articles, editorials, conference abstracts without full text, single case reports, and animal-only studies were not considered eligible. Studies in which RLS participants were not clearly defined or where biomarker results for RLS could not be distinguished from other conditions were also excluded. Research articles exclusively focused on the measurement of ferritin, serum iron, and total iron-binding capacity without their correlation to a novel biomarker were excluded as well. Automated filtering tools provided by the respective databases—exclusion of predefined publication types such as reviews, editorials, and conference abstracts, single case reports, and letters to the editor—were applied during initial screening to remove non-eligible records before manual assessment. The process of study selection is summarized using a PRISMA flow diagram (Figure 2).

### 2.3. Data Charting

A standardized data-charting form was developed by two reviewers (K.A. and T.G.) to define the specific information that was extracted from the approved articles. The following characteristics were independently extracted by both reviewers: (1) author(s) and year of publication; (2) study population characteristics, including number of restless legs syndrome patients and control subjects; (3) inclusion and exclusion criteria; (4) type of serum or plasma biomarkers analyzed and analytical methods used; (5) main statistically significant findings related to biomarker levels; and (6) the authors’ conclusions, including the relevance of the findings to the pathophysiology, diagnosis, or severity of RLS. The data-charting form was repeatedly refined throughout the review process, and discrepancies between reviewers were resolved through discussion and consensus to ensure accuracy and completeness of the extracted data (Table 1, Table 2 and Table 3). Descriptive methodological characteristics underlying the summarized findings are provided in detail in the Supplementary Materials to preserve the clarity and readability of the main table.

## 3. Results

### 3.1. Selection of Sources

The initial database search identified a total of 1050 records, including 495 from Scopus, 227 from PubMed, and 328 from Web of Science. After application of automated tools provided by the search engines of the databases, review articles were removed, rendering only 559 records for screening. Following title and abstract screening, 199 records were excluded because they did not meet the predefined eligibility criteria, including lack of relevance to restless legs syndrome, absence of serum or plasma biomarker analysis, non-human studies, or publication types such as reviews, letters, or editorials.

The full texts of the remaining 195 articles were assessed for eligibility. Of these, 145 studies were excluded because they did not meet the inclusion criteria, including studies that did not analyze serum or plasma biomarkers, focused exclusively on non-peripheral biomarkers (e.g., cerebrospinal fluid or genetic polymorphisms without protein or metabolite measurements), did not expand the explored biomarkers beyond classical iron status assessment, or did not provide sufficient methodological or statistical detail. Ultimately, 50 studies met the eligibility criteria and were included in the final scoping review.

The included studies were further categorized according to biomarker classes, including inflammatory markers (C-reactive protein (CRP) and related inflammatory mediators) and cytokines, iron-regulatory biomarkers (hepcidin), omics-derived biomarkers (proteomic and metabolomic markers), and miscellaneous biomarkers encompassing a wide range of putative novel biomarkers (Figure 2).

### 3.2. Characteristics of Sources and Results from Individual Sources

The included studies were systematically analyzed and grouped according to the type of serum or plasma biomarkers investigated. The first group comprised studies evaluating inflammatory and immune-related biomarkers, including acute-phase proteins and markers of systemic inflammation. The second group included studies assessing cytokines with diverse relations to inflammation. The third group examines articles focusing on an iron-regulatory protein—hepcidin—and the other parameters of iron status. The fourth group consisted of omics-based studies, including proteomic, metabolomic, glycomic, and transcriptomic analyses aimed at identifying novel biomarker signatures associated with RLS. The fifth group included studies investigating miscellaneous biomarkers, such as oxidative stress markers, neurodegeneration-related proteins, neurotrophic factors, metabolic biomarkers, and extracellular vesicle-derived markers.

For each study, key methodological and clinical characteristics were extracted and summarized, including study population demographics, inclusion and exclusion criteria, biomarker type and analytical methods, main statistically significant findings, and conclusions regarding the relevance of the biomarkers to RLS pathophysiology, diagnosis, or disease severity (Table 1, Table 2 and Table 3).

In the Supplementary Materials, the full table, including all the articles chosen for the scoping review, can be found. We decided to include in the main body of the manuscript only the tables on hepcidin, high-throughput omics, and miscellaneous biomarkers. This is encouraged by the fact that there already exists literature on systematic analysis of inflammatory markers [61] and a concise review on cytokines in RLS [62].

### 3.3. Synthesis of Results

After screening articles and applying eligibility criteria, 50 unique studies evaluating serum/plasma (and closely related peripheral) biomarkers in restless legs syndrome were included in the synthesis of results. The processing of evidence revealed a clear methodological shift over the past decade, moving from predominantly targeted panels—iron indices, CRP, and selected cytokines [61,62]—to system-level discovery approaches like various high-throughput omics technologies [37,38,39,40,41,42,52]. Most studies were cross-sectional case–control investigations with modest sample sizes and substantial heterogeneity. It was reflected in the variability in case definition—including idiopathic and secondary RLS, community or symptom-based recruitment. A smaller subset of studies used large population datasets and disease-enriched clinical cohorts, particularly in hemodialysis [35,47,53,63,64], where background inflammation and altered iron trafficking are intrinsic to the host state. Some studies focused on binary contrasts between RLS and controls. Others attached biomarker associations to quantitative traits such as symptom severity and periodic limb movement burden [38,65], as well as to characteristics such as sleep quality and quality of life.

Biomarker signals clustered into recurring themes. These included iron handling and iron-adjacent proteins extending well beyond the simplistic “low ferritin” narrative; immune activation and acute-phase biology spanning CRP [47,63,65,66,67,68,69,70,71,72,73,74], cytokines [65,69,72,75,76,77,78,79], complement, and inflammatory proteomic signatures [38,39,40,41]; oxidative and nitrosative stress [47] with consistent alterations in redox homeostasis; emerging neurotrophic and neuroaxonal injury signals detectable in blood [48,56]; and an expanding set of omics-derived signatures implicating complement/coagulation–kinin pathways [39,40,41,50], IL-17/NF-κB programs [37], and metabolic patterns compatible with uremic-toxin [51,53]. Importantly, these domains do not appear independent; many findings suggest that iron biology is embedded within immuno-inflammatory and redox systems, and that the detectability of peripheral signals is enhanced when analyses are linked to objective motor burden—PLMI or severity rather than diagnosis alone [65].

#### 3.3.1. Beyond Classical Iron Status—Hepcidin as Master Regulator of Iron Metabolism

Although the scope of this review is explicitly “beyond iron,” iron biology remained interwoven through the results. Eleven studies explicitly reported iron indices or iron-related proteins, and the dominant pattern was not uniform deficiency but context-dependent directionality. In idiopathic case–control cohorts, iron and ferritin tended to be lower in RLS [32,34,38,70,71,73,74,79], and in at least one dataset these reductions tracked severity gradients, reinforcing the classical paradigm. In contrast, cohorts enriched for systemic inflammation or comorbidity often showed higher ferritin alongside evidence of impaired bioavailable iron, consistent with ferritin functioning as an acute-phase reactant and with patterns of functional iron deficiency [35,47]. This divergence is important in hemodialysis cohorts, where inflammatory activation and disordered iron utilization coexist, complicating the interpretation of ferritin as a marker of iron stores. In PLMI-oriented proteomics, decreases in hepcidin and ferritin (including ferritin light chain) emerged among the strongest signals, suggesting that regulatory components of iron handling may relate more closely to motor phenotype than conventional ferritin thresholds capture [38]. Overall, the iron-related literature does not reduce to “low ferritin.” It repeatedly indicates state-dependent dysregulation of iron handling shaped by inflammatory context, comorbidity and clinical intensity.

Evidence across seven case–control studies demonstrates dysregulation of hepcidin in RLS [30,31,32,33,34,35,36]. Drug-free patients with primary RLS exhibit significantly elevated serum hepcidin levels compared with controls, even in the presence of normal ferritin concentrations, suggesting altered iron regulatory signaling independent of systemic iron deficiency [32]. Elevated hepcidin levels were associated with disease severity, periodic limb movements, and clinical phenotype, supporting its relevance as a marker of disease activity [33]. Similarly, studies in idiopathic RLS populations confirmed higher hepcidin concentrations despite normal iron and ferritin values, reinforcing the hypothesis that functional iron deficiency and impaired iron bioavailability—not absolute iron depletion—may contribute to pathogenesis [34]. Longitudinal evidence further suggests that reductions in serum hepcidin correlate with clinical improvement following dopaminergic therapy [30]. This suggests a viable role of hepcidin in treatment response monitoring [30]. In secondary RLS populations, particularly those with end-stage renal disease or undergoing dialysis, hepcidin levels were markedly elevated and independently associated with RLS presence and severity [35]. Serum hepcidin also emerged as an independent predictor of RLS in peritoneal dialysis cohorts, alongside markers of inflammation and altered iron utilization [36]. These findings support the hypothesis for a mechanistic link between inflammation-driven hepcidin up-regulation and impaired iron transport across the blood–brain barrier [80,81]. However, population-based studies in healthy cohorts have yielded inconsistent results, with some large-scale analyses finding no independent association between plasma hepcidin and RLS prevalence [31], suggesting potential heterogeneity based on disease subtype, population characteristics, or methodological differences.

#### 3.3.2. Inflammation and Cytokines

Inflammation and immune activation emerged as the most frequently represented “beyond iron” signal. Fourteen studies reported inflammatory and immune markers, encompassing CRP and acute-phase indices, targeted cytokines, immune-related proteomic signatures, and immune-enriched transcriptomic programs. While individual cytokine results varied, a coherent pattern appeared in which inflammatory associations strengthened when associated with PLMI burden. Several studies have reported higher CRP levels or a “high-CRP state” in RLS [47,65,71,72,73]. PLMI correlated with CRP in one dataset, and individuals with very high PLMS burden had substantially higher odds of elevated CRP after multivariable adjustment, whereas IL-6 and TNF-α were not consistently linked to PLMI in that same cohort [65]. Adjusted analyses identified an association between RLS and high CRP. In contrast, other markers of generalized inflammation, such as soluble urokinase-type plasminogen activator receptor (suPAR), did not show similarly robust associations after adjustment [72], showing that not all inflammatory surrogates behave equivalently. CRP signals were not universal in a depression-enriched population; TNF-α was associated with RLS symptoms while CRP was not [69].

Targeted cytokine studies were fewer but more mechanistically explicit, repeatedly implicating IL-6 and TNF-α. In one study, IL-1β, IL-6, and TNF-α were elevated in RLS compared with controls even though hsCRP did not differ, indicating that cytokine activation may be detectable in the absence of a strong CRP signal [78]. In another, IL-6 and TNF-α were increased—the former remained independently associated with RLS after adjustment, and it correlated with symptom severity [76]. In hemodialysis contexts, elevations in hsCRP and IL-6 were again observed, consistent with the expectation of chronic inflammation in this population and supporting the need to acknowledge secondary RLS phenotypes [47]. Transcriptomic analyses revealed enrichment of IL-17 and NF-κB-related programs alongside immune activation and regulated cell death/mitochondrial quality control pathways [37]. PLMI-linked proteomics similarly identified increases in immune mediators such as RANTES/CCL5, and RLS-specific increases included IL-17A [38], aligning with IL-17 axis involvement observed in transcriptomic enrichment. Four of the studies, however, did not show significant associations between selected cytokines and RLS patients [62,65,72,79]. These discrepancies in the findings arise from the methodological inhomogeneity in laboratory methods, cohort characteristics, sample size, etc. The inflammation-oriented research can provide many viable targets for biomarkers, but the results and their interpretations should be treated cautiously. While low-profile immune activation may act as a central domain in RLS, the research is far from establishing a causative relationship.

#### 3.3.3. Oxidative Stress and Other

Oxidative and nitrosative stress presented as another pillar alongside immune activation [41,46,47,51]. The dominant pattern was a shift toward oxidative stress, coupled with depletion of antioxidant buffers; these changes correlated with symptom burden. A classic oxidative profile was reported with decreased nitric oxide and thiols and increased advanced oxidation protein products and malondialdehyde, reflecting both protein oxidation and lipid peroxidation alongside antioxidant depletion [46]. Redox imbalance was corroborated by studies of thiol/disulfide homeostasis, which demonstrated reduced native and total thiol levels and increased disulfide fractions; importantly, disulfide indices correlated with age and RLS severity scales [46,57]. In hemodialysis cohorts, oxidative DNA damage, as indicated by increased 8-OHdG, independently predicted severity, and inflammatory markers were simultaneously elevated, suggesting coupled immuno-redox activation rather than isolated oxidative stress [47]. Omics studies provided mechanistic continuity, with proteomic pathway analyses linking protein shifts to immune activation and oxidative stress. Transcriptomic enrichment implicates ferroptosis and mitophagy programs, which are compatible with oxidative injury and iron–redox coupling [37,38,39,40]. These results support redox imbalance as a robust candidate domain and one of the more clinically relevant biomarker families because associations with severity and motor burden speak directly to disease burden rather than diagnosis alone.

#### 3.3.4. Neurotrophic Factors

Neurotrophic factors and neuroaxonal injury markers constitute a smaller but clinically provocative evidence cluster that, if replicated, would meaningfully broaden the conceptualization of RLS pathobiology. Circulating brain-derived neurotrophic factor (BDNF) showed a directionality consistent with reduced neurotrophic support in selected phenotypes: in Parkinson’s disease and RLS, BDNF was lowest and inversely associated with severity, suggesting either additive neurobiological stress or phenotype-specific mechanisms in a neurodegeneration-enriched context [48,56]. Extending beyond neurotrophic signals, newer work reported increased glial fibrillary acidic protein (GFAP) and neurofilament light chain (NfL) alongside decreased BDNF in RLS compared with controls. With NfL correlating with symptom severity and QoL impairment, and GFAP correlating with QoL impairment [48]. These findings imply that at least some individuals with RLS exhibit measurable peripheral correlates of glial activation and neuroaxonal stress. Neurodegeneration-adjacent overlap was further suggested by reports of increased α-synuclein in specific at-risk contexts, pointing to potential mechanistic intersections with synucleinopathies in selected populations rather than generalizable idiopathic RLS biology [45]. Overall, this domain is compelling but under-replicated and vulnerable to confounding.

#### 3.3.5. Omics

Peripheral blood transcriptome profiling has demonstrated extensive molecular dysregulation in patients with RLS, with over 12,000 genes differentially expressed compared to controls, including thousands of significantly upregulated and downregulated transcripts. Pathway enrichment analyses revealed involvement of multiple interconnected biological networks—infection-related, inflammatory, immunological, metabolic, and neurodegenerative processes [37]. These findings provide compelling evidence that RLS is associated with systemic transcriptional activation of inflammatory cascades such as interleukin-17, nuclear factor-κB, and mitogen-activated protein kinase signaling pathways, which influence neurotransmission, synaptic plasticity, and neuronal integrity [37]. This transcriptomic profile supports the concept that inflammation may serve as a central upstream mechanism linking peripheral molecular dysregulation with CNS dysfunction in RLS. Proteomic analyses of serum and plasma consistently demonstrate differential expression of proteins involved in immune regulation, complement activation, oxidative stress, and proteolytic activity. Early plasma proteomic studies identified increased levels of inflammatory and immune-related proteins, including alpha-1-acid glycoprotein, haptoglobin, complement C4-A, and immunoglobulin components, alongside reduced complement C3 and alpha-1-antitrypsin [39]. The progressive elevation of immunoglobulin levels correlated with disease severity, suggesting a potential relationship between immune activation and clinical progression [39]. Similarly, targeted serum proteomics using liquid chromatography–mass spectrometry revealed significant increases in proteins such as apolipoprotein C-II, leucine-rich alpha-2-glycoprotein-1, and extracellular matrix protein-1, while proteins including alpha-1-antitrypsin, vitamin D-binding protein, and complement-related factors were decreased [41]. These alterations were strongly associated with inflammatory, immune, and oxidative stress pathways, reinforcing the central role of systemic inflammatory dysregulation in RLS pathophysiology [41]. Network-based bioinformatic proteomic analyses have identified complement proteins C3 and C4A, alpha-2-HS glycoprotein, and alpha-2-macroglobulin as central hub molecules linking inflammation, iron metabolism, and hypoxic signaling pathways [40]. These findings suggest that complement activation and immune signaling may contribute to disease pathogenesis by modulating iron homeostasis and neuronal function [40]. Large-scale plasma proteomics studies involving over 2000 individuals have further refined this molecular signature [38]. Multivariate analyses identified reduced levels of iron-related proteins such as hepcidin and ferritin alongside increased catabolic and inflammatory mediators, including RANTES, cathepsin A, and interleukin-17A [38]. These results indicate that although iron dysregulation remains a core feature of RLS, proteomic evidence also highlights involvement of inflammatory cytokines, proteolytic enzymes, and cellular stress response pathways. Metabolomic analyses provide complementary evidence of systemic biochemical alterations in RLS. In patients with peritoneal dialysis-associated RLS, four metabolites—hippuric acid, phenylacetylglutamine, N,N,N-trimethyl-L-alanyl-L-proline betaine, and threonic acid—were significantly altered compared to dialysis controls without RLS [42]. These metabolites demonstrated strong diagnostic performance when combined into a biomarker panel, with area-under-the-curve values exceeding 0.9, indicating excellent discriminative capacity. Many of these metabolites are related to gut microbiota metabolism, oxidative stress, and renal clearance pathways, suggesting that systemic metabolic dysfunction contributes to the pathogenesis of secondary RLS.

#### 3.3.6. Miscellaneous

Across the 19 studies summarized in the table, a common theme is that idiopathic and secondary restless legs syndrome involve a constellation of metabolic, inflammatory, and neurochemical alterations rather than a single biomarker (Table 3). RLS patients exhibited lower serum vitamin B12 levels, and reduced B12 independently predicted RLS and correlated inversely with symptom severity and depression scores [43]. Telomere length was similar across RLS, narcolepsy, obstructive sleep apnea, and control groups (insomnia patients had shorter telomeres) [44], and both leucine-rich repeat kinase 2 and vitamin D receptor gene variants showed no significant association with idiopathic RLS [45,49]. In a separate cohort, serum copper, magnesium, selenium, and calcium were significantly higher in RLS patients, with no correlation with age at onset or disease severity [54]. Lower uric acid levels were reported in RLS patients in another study [51].

Markers of inflammation, oxidative stress and iron metabolism showed more consistent differences. RLS patients had elevated malondialdehyde and advanced oxidation protein products with reduced nitric oxide and thiol concentrations [46]. Those on hemodialysis displayed higher hs-CRP, IL-6, ferritin, NT proBNP and DNA oxidation marker 8-OHdG, lower transferrin saturation, and regression analyses identified hs CRP and 8- OHdG as independent risk factors [47]. Serum thiol/disulfide homeostasis was shifted toward oxidation (increased disulfide and disulfide/thiol ratios), providing high sensitivity and specificity for RLS diagnosis, while uric acid levels were reduced and did not vary with disease severity. In hemodialysis patients, homocysteine correlated positively with RLS occurrence and poor sleep quality, and higher parathyroid hormone levels were linked with worse sleep quality [53]. Serum glial fibrillary acidic protein (GFAP) and neurofilament light chain were elevated, and brain-derived neurotrophic factor (BDNF) decreased, each independently predicting RLS—neurofilaments correlated with symptom severity while BDNF correlated negatively [48]. Similar findings were reported in Parkinson’s disease patients with RLS, where BDNF levels were significantly lower and inversely correlated with severity.

Neurochemical and hormonal studies suggest dysregulation of dopaminergic and circadian systems [55,58]. Peripheral blood analyses revealed higher plasma dopamine in medicated RLS patients and a down-regulation of dopamine D2 receptors on lymphocytes and monocytes, which persisted despite dopaminergic therapy. Constant routine experiments showed that leg discomfort and periodic limb movements followed a circadian rhythm, peaking around 03:00 and coinciding with rising melatonin. Melatonin secretion preceded symptom exacerbation by ~2 h and exogenous melatonin worsened symptoms, whereas bright light (melatonin suppression) improved them [58]. Plasma copeptin, a stress-related vasopressin surrogate, was elevated in RLS but unrelated to symptom severity [59]. Finally, neuronal extracellular vesicle studies revealed increased total and heavy chain ferritin in RLS, correlating with systemic iron indices but not with brain iron load [60].

## 4. Discussion

### 4.1. Summary of Evidence

The available evidence from molecular biomarker studies indicates that restless legs syndrome is associated with measurable biological alterations. These findings support a model in which RLS is associated with interactions between iron handling, immune signaling, and neuronal regulation. Inflammatory markers show a recurring pattern of mild systemic immune activation in at least a subset of patients with RLS. This is something that has long been assumed by Weinstick et al. [82]. According to this theoretical review, 95% of the 38 highly associated RLS conditions named in the article are also associated with inflammatory changes [82]. Cytokines such as interleukin-6, tumor necrosis factor-α, and interleukin-1β have been found at higher concentrations in some RLS populations. These molecules are known to influence iron metabolism, suggesting that inflammatory signaling may contribute to physiological conditions that affect neuronal excitability and sensorimotor integration. This is especially true for IL-6 since it is well recognized that this cytokine provokes the synthesis and release of hepcidin by the liver, acting as a focal point in the link between iron metabolism and the immune system. Consistency of these findings varies between cohorts due to biological heterogeneity rather than a uniform inflammatory state. Hepcidin regulates ferroportin activity and therefore determines whether iron remains sequestered in storage sites or becomes available for cellular use [26]. Some studies report elevated hepcidin levels in RLS, especially in populations with comorbid conditions such as chronic kidney disease. In such a scenario, iron could remain sufficient at the systemic level while being less available to neural tissues that rely on tightly regulated iron transport mechanisms [80]. Observations that hepcidin levels may change with treatment and correlate with symptom changes suggest that iron regulatory dynamics may reflect disease activity in certain contexts [30].

Proteomic and metabolomic analyses provide robust evidence of systemic biological differences between individuals with and without RLS. Several studies identify altered expression of proteins involved in immune regulation, oxidative balance, and molecular transport. While these findings do not identify a single defining pathway, they support the presence of broader physiological perturbations. Glycomic and extracellular vesicle analyses further suggest that intercellular signaling and protein modification patterns differ in RLS [52,60].

Markers associated with neuronal integrity and physiological stress have also been investigated. Elevated neurofilament light chain and glial fibrillary acidic protein levels in some cohorts suggest subtle alterations in neuronal or glial homeostasis [48]. Reduced brain-derived neurotrophic factor in certain studies may indicate differences in neuronal adaptability or plasticity. These changes could play a role in the altered neurotransmission in RLS.

**Figure 3:**
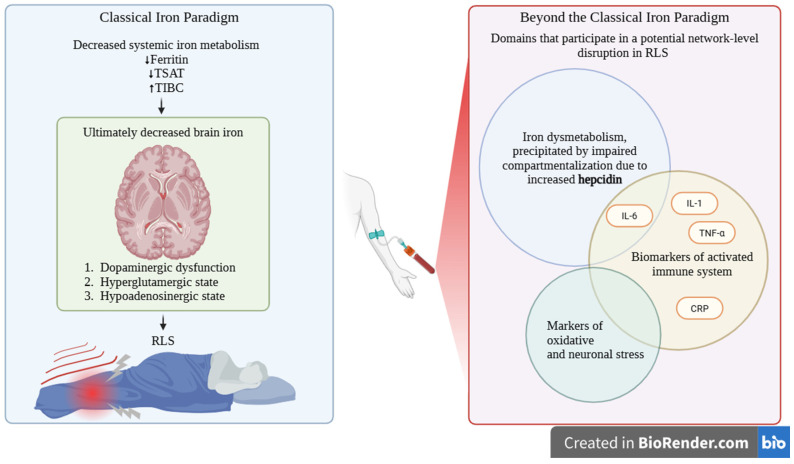
Classical versus expanded pathophysiological model of restless legs syndrome (RLS). (**Left**): Systemic iron deficiency leads to reduced brain iron, causing dopaminergic dysfunction, hyperglutamatergic activity, and hypo-adenosinergic signaling, resulting in RLS. (**Right**): An expanded model integrates hepcidin-mediated iron dysmetabolism with immune activation and oxidative/neurostress, reflecting a network-level pathophysiology. RLS—restless legs syndrome; TSAT—transferrin saturation; TIBC—total iron-binding capacity; IL-6—interleukin-6; IL-1—interleukin-1; TNF-α—tumor necrosis factor alpha; CRP—C-reactive protein.

Importantly, the identified biomarker domains do not operate independently. Iron dysregulation appears embedded within inflammatory signaling networks. This potentially occurs within the axis of interleukin 6—hepcidin modulation of iron metabolism. Oxidative stress and immune activation may form a coupled system, where redox imbalance both results from and amplifies inflammatory processes. This interdependence suggests that RLS is better conceptualized as a network-level disorder (Figure 3).

### 4.2. Limitations

This scoping review has several limitations inherent both to the scoping review methodology and to the specific strategies employed. By design, scoping reviews aim to map the breadth of existing literature rather than to perform formal quantitative synthesis or risk-of-bias assessment. The present review does not include a systematic evaluation of study quality, and the findings should be interpreted as an overview of emerging biological domains rather than definitive evidence supporting specific biomarkers. The review was restricted to articles published in English, which introduces the possibility of language bias. Gray literature, conference abstracts without full text, and unpublished studies were not included, which may contribute to publication bias and incomplete capture of available evidence. A major limitation of the current evidence base is the lack of consistent replication across independent cohorts, particularly for emerging biomarkers identified through omics approaches. Furthermore, the majority of included studies are cross-sectional and observational, limiting causal inference. A considerable part of the reported associations should be interpreted as hypothesis-generating rather than indicative of mechanistic relationships. Although the search strategy included three major biomedical databases (PubMed, Scopus, and Web of Science) and was designed to maximize sensitivity, it remains possible that relevant studies indexed in other databases or published outside indexed journals were not identified.

### 4.3. Conclusions

This scoping review attempts to capture the breadth of existing literature on the topic of serum biomarkers in RLS, while targeting specifically the novel lanes of research beyond the classical iron–ferritin paradigm. Despite the apparent divergence of serum biomarker research in restless legs syndrome into multiple exploratory trajectories, three dominant biological nexuses consistently emerge: a low-grade inflammatory milieu, aberrant hepcidin-dependent iron regulatory signaling, and oxidative stress coupled with neuronal stress. The most consistent associations occur when RLS is linked to objective motor burden or severity. Periodic limb movements during sleep should be considered to take a more central place among the diagnostic criteria and not simply function as an adjunct. Inflammatory signaling may have the property to track motor burden intensity. RLS diagnosis is phenomenological, potentially biologically outdated, and due for objective stratification. The future of RLS diagnosis will likely depend not on refining interview questions, but on identifying measurable biomarkers and network-level dysfunctions that distinguish the true disease entities.

## Figures and Tables

**Table 1:** Studies examining the changes in hepcidin levels in patients with RLS.

Authors	Study Subjects	Main Results	Conclusions
Im et al. 2020 [30]	18 non-anemic, drug-naive idiopathic RLS patients vs. 15 healthy controls; 12 patients reassessed after 12-wk pramipexole	Baseline serum hepcidin was similar between groups; after pramipexole therapy, a greater fall in hepcidin correlated with a larger improvement in IRLS score and QoL; baseline hepcidin was not predictive.	Serum hepcidin decrease predicts treatment response in idiopathic RLS.
Dowsett et al. 2021 [31]	Large population-based cohort of 9708 healthy blood donors (466 RLS cases vs. 9242 controls)	Plasma hepcidin levels were identical in cases and controls (~10.5 ng/mL); logistic regression was adjusted for age, sex, alcohol, smoking, etc., and showed no association (OR ≈ 1.00 per 1 ng/mL increase).	No evidence that plasma hepcidin predicts RLS; limitations: diurnal variability of hepcidin (samples collected at different times), plasma rather than serum measurement and few severe RLS cases.
Dauvilliers et al. 2018 [32]	In total, 108 drug-free primary RLS patients vs. 45 controls with normal ferritin.	Serum hepcidin and hepcidin/ferritin ratio were higher in RLS patients; hepcidin was positively correlated with periodic leg movements during sleep/wakefulness; a U-shaped relation existed between hepcidin and RLS severity.	Hepcidin may be a prognostic biomarker, but results should be interpreted with caution.
Chenini et al. 2020 [33]	In total, 102 drug-free moderate-to-very-severe RLS patients vs. 73 community controls; 34 patients reassessed under dopaminergic therapy.	Ferritin was slightly higher in the RLS group but nonsignificant after BMI adjustment; serum hepcidin was higher in RLS patients (median 18.36 µg/L vs. 11.89 µg/L) and remained higher after excluding low ferritin cases; ROC showed a hepcidin cut-off of 18.1 µg/L (sensitivity 52%, specificity 75%); hepcidin correlated with age, BMI and periodic leg movements and was unaffected by dopaminergic therapy.	Hepcidin appears more informative than ferritin as a biomarker; hepcidin levels are unaffected by treatment or augmentation.
Alaçam Köksal et al. 2023 [34]	In total, 40 idiopathic RLS patients vs. 40 age-/sex-matched healthy controls.	RLS patients had higher red-blood-cell count, neutrophil count and hepcidin levels; ferritin and iron were similar across groups; logistic regression identified hepcidin as an independent predictor, and an ROC cut-off of 10.45 ng/mL predicted RLS (sensitivity 87.5% and specificity 55%).	Elevated hepcidin supports its role in RLS pathogenesis.
Tufekci & Kara 2021 [35]	In total, 72 chronic hemodialysis patients (36 with RLS vs. 36 without).	RLS patients had higher serum hepcidin, HbA1c and ferritin; hepcidin positively correlated with IRLS severity; multivariate regression identified hepcidin and HbA1c as independent predictors of RLS; hepcidin cut-off at ~165 ng/mL predicted RLS with a sensitivity of 61% and specificity of 78%.	Increased hepcidin associated with RLS in hemodialysis patients; uncontrolled diabetes contributes to this.
Guo et al. 2021 [36]	In total, 51 peritoneal dialysis (PD) patients with RLS vs. 102 PD patients without RLS.	The RLS group showed longer PD duration, higher serum hepcidin and calcium, and lower hemoglobin, albumin and residual kidney function; logistic regression identified PD duration (OR ≈ 1.04), hemoglobin, calcium, albumin, hepcidin (OR ≈ 1.02) and residual kidney function as independent determinants; hepcidin > 105.78 ng/mL predicted RLS.	Hepcidin is associated with RLS in PD patients and may serve as a positive predictive biomarker.

**Table 2:** Studies involving the application of high-throughput omics in RLS patients.

Authors	Study Subjects	Main Results	Conclusions
Mogavero et al., 2024 [37]	In total, 17 adults with RLS and 18 drug-free controls had venous blood drawn after a 12 h fast. RNA from peripheral blood mononuclear cells was profiled using next-generation sequencing.	Differential expression analysis identified 12,857 transcripts (4932 up-regulated and 3971 down-regulated) between RLS and controls. Enrichment analysis highlighted nine dysregulated network groups (infections, inflammation, immunology, neurodegeneration, cancer, neurotransmission, blood and metabolic mechanisms). Many genes were related to IL-17, transient receptor potential channels, NF-κB, NOD-like receptor, MAPK, p53, mitophagy and ferroptosis signaling.	The authors concluded that genetic predisposition and inflammatory/immune pathways involving mainly neurotropic viruses and TORCH complex may trigger RLS.
Cederberg et al., 2024 [38]	In total, 1410 adults from the STAGES cohort (102 screened positive for RLS) underwent plasma proteomic profiling; replication used 697 subjects from a second sleep-clinic cohort (combined n = 2 107).	Multivariate analyses identified 68 proteins associated with the PLM index. Iron-related proteins (hepcidin, ferritin and ferritin light chain) were decreased, and inflammatory/lysosomal proteins (RANTES, cathepsin A, and SULT1A3) were increased. In the combined dataset, the top proteins were hepcidin, cathepsin A, ferritin and RANTES. Comparing RLS vs. controls identified cathepsin Z, heme-oxygenase 2 and interleukin-17A.	The study confirmed that PLM is associated with low iron status and suggested involvement of proteolytic enzymes (e.g., cathepsin A) in PLM/RLS. The authors recommended hepcidin, ferritin and cathepsin A as biomarkers for PLM and emphasized that results for RLS were more variable because few subjects had RLS.
Bellei et al., 2018 [39]	Plasma from 34 patients with primary RLS (17 with mild–moderate symptoms and 17 with severe/very severe symptoms) and 17 age- and sex-matched controls was analyzed using 2D gel electrophoresis coupled with LC-MS/MS.	Five proteins (α-1B-glycoprotein, α-1-acid glycoprotein 1, haptoglobin, complement C4-A and immunoglobulin κ constant) were up-regulated in both RLS groups vs. controls. High-severity RLS showed additional increases in kininogen-1, immunoglobulin heavy constant α1 and immunoglobulin λ constant 2 and decreases in α-1-antitrypsin. Complement C3 was down-regulated in all patients.	The authors concluded that plasma proteins associated with inflammation and immune response may underlie RLS; gradual increases in immunoglobulins correlated with symptom severity. They suggested that under-expression of α-1-antitrypsin and up-regulation of kininogen-1 could signal cardiovascular risk in severe RLS.
Shin et al., 2020 [40]	Serum from 7 drug-naïve idiopathic RLS patients (30–45 y) and 6 age- and sex-matched healthy controls was analyzed by 2D electrophoresis and MALDI-TOF/TOF MS.	Eight differentially expressed proteins were identified. Complement C3, β-2-glycoprotein I (APOH), inter-α-trypsin inhibitor heavy chain 4 and vitamin D binding protein were up-regulated, whereas complement C4A, coagulation factor XII, α-2-HS glycoprotein (AHSG), and α-2 macroglobulin (A2M) were down-regulated. Network analysis showed that C3, C4A, AHSG and A2M occupy hub positions and are linked to iron-deficiency and inflammation pathways.	The study suggested that complement proteins (C3 and C4A) and acute phase proteins (A2M and AHSG) may serve as biomarkers reflecting iron deficiency and inflammatory processes in RLS. Western blot validation confirmed increased C3 and decreased C4A.
Mondello et al., 2021 [41]	Serum from 12 patients with clinical RLS (mean age ≈ 68 y; 8 women) and 10 healthy controls (mean age ≈ 68 y) was analyzed by LC-MS/MS.	Proteomic profiling quantified 272 proteins; 243 were shared between groups. Five proteins (apolipoprotein C-II, leucine-rich α-2-glycoprotein 1, FLJ92374, extracellular matrix protein 1 and FLJ93143) were markedly increased, while nine proteins (vitamin D binding protein, FLJ78071, α-1-antitrypsin, CD5 antigen-like, haptoglobin, fibrinogen α chain, complement factor H-related protein 1, platelet factor 4 and plasma protease C1 inhibitor) were decreased in RLS.	The authors concluded that RLS is a multifactorial disorder involving immune, inflammatory, and oxidative pathways and proposed the identified protein panel as potential biomarkers and therapeutic targets.
Yang et al., 2022 [42]	Peritoneal dialysis (PD) patients: discovery set included 27 PD patients with RLS and 30 PD patients without RLS; validation set comprised 51 PD-RLS and 51 PD controls.	Non-targeted UPLC-Q-TOF/MS metabolomics identified 32 differential metabolites; four (hippuric acid, phenylacetylglutamine, N,N,N-trimethyl-L-alanyl-L-proline betaine, and threonic acid) were consistently altered in both discovery and validation sets. A biomarker panel combining these four metabolites achieved an area-under-the-curve > 0.9 for distinguishing PD-RLS from PD controls.	The authors suggested that the four-metabolite panel has good diagnostic and predictive ability for PD-associated RLS and that metabolomics can aid early diagnosis and treatment. They did not explicitly discuss limitations, but the findings are based on PD patients and may not generalize to idiopathic RLS.

**Table 3:** Studies targeting the exploration of novel biomarkers that were not reproduced.

Authors	Study Subjects	Main Results	Conclusions
Geng et al., 2022 [43]	In total, 80 RLS patients vs. 80 age-/sex-matched controls. Cross-sectional retrospective study.	RLS patients had lower serum vitamin B12 and albumin and higher creatinine and homocysteine vs. controls. Serum vitamin B12 inversely correlated with RLS severity and depression scores; logistic regression showed reduced B12 was independently associated with RLS.	Vitamin B12 deficiency is associated with RLS and symptom severity; suggests monitoring B12 in RLS management.
Eşel et al., 2025 [44]	In total, 94 patients; RLS subgroup: 21 patients (14 F/7 M), mean age ~39 yr; controls matched for age/sex.	Telomere length measured by RT-PCR. No significant difference in telomere length between patient groups and controls; the insomnia group had shorter telomeres than the RLS and OSAS groups. Telomere length was positively correlated with sleep efficiency; no association with RLS severity.	Telomere shortening is not a biomarker for RLS.
Guo et al., 2025 [45]	In total, 199 idiopathic RLS patients, 114 Parkinson’s disease patients, 273 healthy controls.	Frequencies of LRRK2 variants examined. The G2385R variant is frequent in Parkinson’s disease but not in RLS; logistic regression showed only marginal association of LRRK2 mutations with RLS (OR ≈ 1.85). Plasma α-synuclein levels were higher in LRRK2 carriers with RLS or Parkinson’s disease vs. non-manifesting carriers.	LRRK2 mutations are strongly associated with Parkinson’s disease but only weakly with RLS; elevated plasma α-synuclein suggests a link between RLS and Parkinson’s disease.
Baskol et al., 2012 [46]	In total, 22 primary RLS patients vs. 20 age-/gender-matched healthy controls.	RLS patients showed lower nitric oxide and thiol levels and higher advanced oxidation protein products (AOPP) and malondialdehyde (MDA) compared with controls. MDA correlated positively with AOPP; thiol was negatively correlated with AOPP.	Increased oxidative stress markers and reduced antioxidants suggest oxidative stress involvement in RLS pathogenesis.
Higuchi et al., 2015 [47]	In total, 159 hemodialysis patients (22% with RLS); cross-sectional multicenter study.	RLS prevalence was 22%. RLS patients had longer dialysis duration; serum hs-CRP, IL-6, ferritin, NT-proBNP and oxidative DNA damage marker 8-OHdG were higher, and transferrin saturation was lower. Regression showed hs-CRP and 8-OHdG as independent risk factors for RLS; 8-OHdG correlated with RLS severity.	Oxidative stress and inflammation are associated with RLS in hemodialysis patients; 8-OHdG may predict RLS severity.
Yan et al., 2025 [48]	In total, 108 newly diagnosed, drug-naïve RLS patients vs. 90 age-/sex-matched healthy controls.	Serum glial fibrillary acidic protein (GFAP), brain-derived neurotrophic factor (BDNF) and neurofilament light chain (NfL) were measured via ELISA. RLS patients had higher GFAP and NfL and lower BDNF vs. controls. NfL correlated positively with symptom severity and quality of life; BDNF negatively correlated with severity.	Elevated GFAP and NfL and reduced BDNF may serve as serum biomarkers for RLS and reflect disease severity.
Jiménez-Jiménez et al., 2021 [49]	In total, 111 RLS patients and 167 controls.	Serum 25-hydroxyvitamin D levels were higher in RLS patients than controls; vitamin D levels were unrelated to age at onset, disease severity or therapy response.	Increased serum vitamin D concentrations may be associated with RLS.
Bellei et al., [50]	In total, 14 high-severity RLS patients vs. 15 healthy controls.	Plasma kininogen-1 (KNG1) and α1-antitrypsin (A1AT) quantified by ELISA. KNG1 levels were significantly higher in high-severity RLS patients, and A1AT levels were lower compared with controls.	Up-regulation of KNG1 and down-regulation of A1AT may link severe RLS to cardiovascular risk; these proteins could be candidate biomarkers for CVD risk in severe RLS.
Yazar et al., [51]	In total, 281 RLS patients vs. 237 age-/sex-matched controls;	RLS patients had lower hemoglobin, ferritin and uric acid levels, and higher total iron-binding capacity (TIBC) than controls. Uric acid levels were not affected by disease severity; variables affecting uric acid were age, disease duration and hemoglobin.	Low serum uric acid, a biomarker of oxidative stress, may contribute to RLS pathogenesis.
Dong et al., [52]	In total, 12 drug-naïve patients with primary RLS vs. 10 healthy controls.	In total, 24 N-glycan structures differed significantly between RLS and controls.	Specific serum N-glycan and isomer alterations may serve as novel biomarkers for RLS and provide insight into disease mechanisms.
Güdel et al., [53]	In total, 52 adults with end-stage renal disease on hemodialysis (26 patients with RLS, 26 age- and sex-matched hemodialysis controls).	Homocysteine levels trended higher in RLS cases, and partial correlation controlling for albumin showed a significant association between homocysteine and RLS occurrence; parathyroid hormone levels were linked with poor sleep quality.	Hyperhomocysteinemia may contribute to uremic RLS; RLS impairs sleep quality in hemodialysis patients.
Yang [42] et al.	In total, 159 peritoneal dialysis (PD) patients: discovery cohort of 57 (30 PD without RLS and 27 PD-RLS)	Untargeted UPLC-Q-TOF/MS metabolomics identified a panel of four metabolites—hippuric acid, phenylacetylglutamine, N,N,N-trimethyl-L-alanyl-L-proline betaine, and threonic acid—that discriminated PD-RLS from PD controls with an area under the curve > 0.9.	The four-metabolite panel may aid early diagnosis of PD-associated RLS.
Jiménez-Jiménez et al. 2022 [54]	Case–control study of 100 idiopathic RLS patients and 110 age- and sex-matched controls (Spanish Caucasians).	Compared with controls, RLS patients had significantly higher serum calcium, copper, magnesium, and selenium; zinc and other elements did not differ. Correlations between trace elements and age at onset or IRLS scores were weak.	Elevated copper, magnesium, selenium, and calcium may be associated with idiopathic RLS, but causality cannot be inferred.
Mitchell et al. [55]	In total, 13 primary RLS subjects (7 on dopaminergic medication, 6 unmedicated) and 12 age-matched healthy controls.	Plasma dopamine was significantly higher in medicated RLS subjects than in unmedicated and control subjects, while epinephrine and norepinephrine did not differ. Flow cytometry revealed down-regulation of dopamine D2 receptor on lymphocytes and monocytes in both medicated and unmedicated RLS subjects vs. controls.	The authors conclude that peripheral D2 receptor down-regulation may serve as a biomarker of RLS and is not normalized by dopaminergic therapy.
Huang et al. [56]	In total, 249 Chinese Parkinson’s disease patients (53 with RLS, 196 without) and 326 age-matched controls.	RLS prevalence in Parkinson’s disease was 21.3%. Serum brain-derived neurotrophic factor (BDNF) was significantly lower in Parkinson’s disease with RLS patients compared with Parkinson’s disease without RLS, controls with RLS, and controls without RLS. BDNF levels negatively correlated with IRLS scores, and regression analyses indicated BDNF as an independent contributor to RLS severity.	Decreased serum BDNF may be involved in RLS pathophysiology in Parkinson’s disease and could serve as a biomarker.
Küçüksayan et al. [57]	Cross-sectional study with 25 primary RLS patients and 25 age- and sex-matched controls.	RLS patients had lower serum total and native thiol levels and higher disulfide levels; disulfide/native thiol and disulfide/total thiol ratios were increased, while the native/total thiol ratio was decreased. ROC analysis showed high sensitivity and specificity of these markers for distinguishing RLS.	Dynamic thiol/disulfide homeostasis reflects oxidative stress and may serve as a diagnostic biomarker for RLS.
Michaud et al. [58]	In total, 7 patients with primary RLS (mean age ~44 years; 3 men, 4 women) and 7 age- and sex-matched healthy controls.	During a 28 h modified constant routine, both groups exhibited circadian rhythms in leg discomfort and periodic leg movements (PLMs). In patients, sensory and motor symptoms peaked around 03:00 h, correlated with low core body temperature and rising salivary melatonin; melatonin secretion preceded symptom exacerbation by about 2 h.	Findings support an intrinsic circadian rhythm of RLS symptoms and implicate melatonin in nocturnal symptom worsening.
Topaloğlu Tuac et al. [59]	In total, 41 primary RLS patients and 41 age- and sex-matched healthy controls.	Plasma copeptin levels were significantly higher in RLS patients (mean 0.93 ng/mL) than in controls (0.46 ng/mL). However, copeptin did not correlate with RLS severity, disease duration, or Epworth Sleepiness Scale scores.	Elevated copeptin suggests hypothalamic–pituitary–adrenal axis activation in RLS. Copeptin may not reflect disease severity.
Chawla et al. [60]	In total, 20 RLS participants and 28 age- and sex-matched controls; participants were screened via polysomnography and excluded for other neurological, psychiatric or sleep disorders.	Neuronal extracellular vesicles (nEVs) were isolated from serum by immunocapture for neural cell adhesion molecule. Participants with RLS had significantly higher total ferritin in nEVs than controls. Western blots revealed that heavy-chain ferritin, but not light-chain ferritin, was elevated in RLS nEVs. nEV ferritin correlated with serum ferritin and other iron parameters in RLS, but not with MRI-measured brain iron deposition.	This first use of nEVs in RLS suggests increased neuronal heavy-chain ferritin export and dysregulation of iron homeostasis. The technique shows promise for biomarker development but is limited by a small sample size.

## Data Availability

No new data were created or analyzed in this study. Data sharing is not applicable.

## Supplementary Materials

The following supporting information can be downloaded at: https://www.mdpi.com/article/10.3390/ijms27083385/s1.

## Abbreviations

The following abbreviations are used in this manuscript:

8-OHdG	8-hydroxy-2′-deoxyguanosine
A1AT	Alpha-1 Antitrypsin
A2M	Alpha-2 Macroglobulin
AHSG	Alpha-2-HS Glycoprotein
AOPPs	Advanced Oxidation Protein Products
APOH	Apolipoprotein H
BDNF	Brain-Derived Neurotrophic Factor
BEST	Biomarkers, EndpointS, and other Tools
BMI	Body Mass Index
C3	Complement Component 3
C4A	Complement Component 4A
CCL5	C-C Motif Chemokine Ligand 5 (RANTES)
CKD	Chronic Kidney Disease
CNS	Central Nervous System
CRP	C-reactive Protein
DNA	Deoxyribonucleic Acid
ELISA	Enzyme-Linked Immunosorbent Assay
FDA	Food and Drug Administration
GFAP	Glial Fibrillary Acidic Protein
hsCRP	High-Sensitivity C-Reactive Protein
IL-1β	Interleukin-1 Beta
IL-6	Interleukin-6
IL-17/IL-17A	Interleukin-17/Interleukin-17A
IRLS	International Restless Legs Syndrome Rating Scale
JBI	Joanna Briggs Institute
KNG1	Kininogen-1
LC-MS/MS	Liquid Chromatography–Tandem Mass Spectrometry
LRRK2	Leucine-Rich Repeat Kinase 2
MALDI-TOF/TOF MS	Matrix-Assisted Laser Desorption–Ionization Time-of-Flight Tandem Mass Spectrometry
MAPK	Mitogen-Activated Protein Kinase
MDA	Malondialdehyde
MEDLINE	Medical Literature Analysis and Retrieval System Online
MRI	Magnetic Resonance Imaging
NF-κB	Nuclear Factor Kappa-B
NfL	Neurofilament Light Chain
NIH	National Institutes of Health
nEVs	Neuronal Extracellular Vesicles
NT-proBNP	N-terminal pro-B-type Natriuretic Peptide
OR	Odds Ratio
PD	Peritoneal Dialysis
PLMs	Periodic Limb Movements
PLMI	Periodic Limb Movement Index
PLMS	Periodic Limb Movements During Sleep
PRISMA-ScR	Preferred Reporting Items for Systematic Reviews and Meta-Analyses Extension for Scoping Reviews
QoL	Quality of Life
RLS	Restless Legs Syndrome
RNA	Ribonucleic Acid
ROC	Receiver Operating Characteristic
suPAR	Soluble Urokinase-Type Plasminogen Activator Receptor
TIBC	Total Iron-Binding Capacity
TNF-α	Tumor Necrosis Factor Alpha
TORCH	Toxoplasmosis, Other infections, Rubella, Cytomegalovirus, Herpes simplex
UPLC-QTOF/MS	Ultra-Performance Liquid Chromatography–Quadrupole Time-of-Flight Mass Spectrometry
